# Low expression levels of putative HPV encoded microRNAs in cervical samples

**DOI:** 10.1186/s40064-016-3524-3

**Published:** 2016-10-22

**Authors:** Elina Virtanen, Tuuli Pietilä, Pekka Nieminen, Kui Qian, Eeva Auvinen

**Affiliations:** 1 Department of Virology, University of Helsinki and Helsinki University Hospital, POB 21, 00014 Helsinki, Finland; 2 Institute of Biotechnology, University of Helsinki, 00014 Helsinki, Finland; 3 Obstetrics and Gynecology, University of Helsinki and Helsinki University Hospital, 00014 Helsinki, Finland; 4 Blueprint Genetics, Helsinki, Finland; 5 Shanghai Genebank Biotechnology Co. Ltd., Shanghai, China

## Abstract

Using small RNA sequencing of libraries established from cervical samples and cervical cancer cell lines, we have previously reported identification of nine and validation of five putative microRNA species encoded by human papillomaviruses (HPV) including five microRNAs encoded by HPV 16. Here we have studied the expression of HPV 16 encoded microRNAs in cervical samples and in HPV 16 containing cell lines. Different sample matrices were collected for the study: 20 paraffin embedded cervical tissue samples, 16 liquid cytology samples, and 16 cervical cell samples from women attending colposcopy due to cervical abnormalities, as well as four HPV 16 containing cell lines. Total RNA was extracted, the samples were spiked with small synthetic control RNAs, and the expression of five HPV 16 encoded microRNAs was assessed by real-time PCR amplification. HPV encoded microRNAs could be frequently detected, albeit at high cycle counts. HPV16-miR-H1 was detected in 3.6 %, HPV16-miR-H3 in 23.6 %, HPV16-miR-H5 in 7.3 %, and HPV16-miR-H6 in 18.2 % of all valid samples. True positive signals for HPV16-miR-H2 could not be detected in any of the samples. Viral microRNAs were detected most frequently in paraffin-embedded samples: in one sample representing normal squamous epithelium, in one cervical intraepithelial neoplasia (CIN) grade 1, one CIN2, three CIN3, two squamous cell carcinoma, three adenocarcinoma in situ, and two adenocarcinoma samples. One liquid cytology sample from a patient with CIN3 as well as all four cell lines were positive for HPV16-miR-H3. In all cases HPV encoded microRNAs were expressed at low levels.

**Electronic supplementary material:**

The online version of this article (doi:10.1186/s40064-016-3524-3) contains supplementary material, which is available to authorized users.

## Background

Human papillomavirus (HPV) infections are the established cause of cervical cancer, and they are implicated in other cancers in various anatomical sites of women and of men. In cervical cancer screening, as well as in triage and follow-up of cervical disease, high-risk (hr) HPV testing is gaining a firm foothold as an alternative to or in parallel with cytology. In addition to positive HPV DNA or mRNA findings, the use of cellular protein markers such as p16^INK4a^ (von Knebel et al. 2012) or DNA methylation markers (Lorincz et al. 2016) have been suggested to improve the specificity of patient management.

MicroRNAs (miRNAs) are small, non-coding regulatory RNA molecules, which regulate the expression of numerous if not most human genes. Many viruses also encode their own miRNAs, and particularly DNA viruses replicating largely in the nucleus have been suggested to encode miRNAs. For example for BK, JC and Merkel polyomaviruses, miRNAs have proven significant in regulating host immune responses and in the establishment of persistent infection (Seo et al. 2008, 2009; Bauman et al. 2011; Lee et al. 2011). Viruses also modify human miRNA expression, and several studies including our own have reported modifications due to HPV (Melar-New and Laimins 2010; Greco et al. 2011; Zheng and Wang 2011), but whether HPV encodes miRNAs of its own has remained a controversial issue. Gu et al. reported a computer prediction of miRNAs encoded by cutaneous and mucosal HPVs (Gu et al. 2011). We have previously published identification and validation of putative HPV encoded miRNAs (Qian et al. 2013). Our work was based on sequencing of small RNA libraries established from human tissue samples representing HPV associated cervical disease, or from HPV containing cell lines. Bioinformatic tools were developed (Qian et al. 2012) and altogether nine HPV encoded miRNAs were predicted. Attempts were made to validate five of these, and four HPV encoded miRNAs were successfully validated: two encoded by HPV 16, one by HPV 38, and one by HPV 68. Among the original RNA sequencing results the HPV16-miR-H1 microRNA had only a few read counts in RNA sequencing while HPV16-miR-H2 read counts were rather high, and both of these were validated by looped-primer RT-PCR and in situ hybridization, suggesting that sequencing read counts cannot be taken as a direct measure of microRNA expression (Qian et al. 2013).

In order to study and validate the expression of all five putative HPV 16 encoded microRNAs, we subjected a set of clinical samples representing HPV associated cervical disease as well as HPV 16 containing cell lines to real-time PCR analysis for viral miRNAs. In this work an LNA enhanced primer based assay was used because the design paradigms of the looped-primer RT-PCR assay used in our previous study (Qian et al. 2013) were not compatible with some of the putative miRNA sequences. The expression of HPV 16 encoded microRNA was fairly common among our sample material, and HPV miRNAs were frequently detected in paraffin-embedded samples and in cell lines. Based on high PCR cycle counts we conclude that the expression levels were altogether low. Although human miRNAs are robustly regulated and may be of use in the diagnosis and monitoring of HPV positive patients, the low expression levels of HPV encoded miRNAs suggest that they are not suitable biomarkers for patient management. However, the frequent detection of HPV encoded miRNAs in HPV positive tissue and in cell lines suggests that viral microRNA may play a role in the regulation of viral replication and pathogenesis.

## Methods

### Samples

Because no established sample type or experimental procedures exist, we attempted microRNA detection from different sample matrices collected for other HPV studies, including formalin-fixed paraffin-embedded archived samples (FFPE) similar to our previous study (Qian et al. 2013). Altogether 52 cervical samples were collected for the study. Twelve cervical samples were collected in Aptima sampling medium (Hologic, Marlborough, MA). Four samples were collected in Hybrid Capture 2 (HC2) specimen transport medium (Qiagen, Gaithersburg, MD). Sixteen samples were liquid-based cytological samples collected in ThinPrep medium (Hologic). Twenty FFPE samples representing different grades of HPV associated lesions were collected from the archives of the Department of Pathology, Helsinki University Hospital Laboratory, and they had all been used in our previous validation study except for sample N:o 39 (Qian et al. 2013). Aptima samples had been stored at −20 °C for ca 12 months, HC2 samples for 2 months, and ThinPrep samples for 2 months. RNA preparations of FFPE samples originated from a previous study and had been stored at −70 °C for ca 2 years. The use of cervical patient samples was approved by the Ethical Committee of the Helsinki and Uusimaa Hospital District.

Four HPV 16 containing cell lines were included in the study as well. HPK IA and HPK II cells were established and provided by Dr. Matthias Dürst (German Cancer Research Center, Heidelberg, Germany; present address: University Clinic Jena, Germany) (Dürst et al. 1987). The cells were established by transfection of primary human foreskin keratinocytes with HPV 16. CaSki epidermoid cervical carcinoma cells and SiHa human cervical tumor cells were purchased from the American Type Culture Collection (ATCC, Manassas, VA). All cells were cultured in DMEM (Sigma-Aldrich Inc., Saint Louis, MO) supplemented with 10 % fetal bovine serum and penicillin/streptomycin at 37 °C and 5 % CO_2_ in a humidified incubator.

### HPV detection and genotyping in clinical samples

All clinical samples were originally collected for other HPV studies, and HPV detection and/or genotyping was performed with the methods used in those studies. These included the Hybrid Capture 2 (HC2) liquid hybridization assay (Qiagen; Virtanen et al. 2016), the Aptima HPV assay (Hologic; Virtanen et al. 2016), the LDR-PCR microarray genotyping assay developed in our laboratory (Qian et al. 2013; Ritari et al. 2012), GeneXpert HPV (Cepheid, Sunnyvale, CA) and the Luminex genotyping assay (Multimetrix/Mikrogen, Neuried, Germany). Of these, the LDR-PCR assay has the highest analytical sensitivity.

### RNA extraction

Aliquots of clinical samples were processed as follows: 600-μl aliquots of Aptima samples, 300-μl aliquots of HC2 samples and 4-ml aliquots of ThinPrep samples were extracted using the miRCURY RNA Isolation Kit—Cell and Plant (Exiqon A/S, Vedbaek, Denmark) applying the preparation protocol for nasal or throat swabs. For HC2 and ThinPrep samples the cells were first centrifuged down (5 min 12,000*g* for HC2 and 15 min 2900*g* for ThinPrep), after which the supernatant was removed and 600 μl of lysis solution was added. After 5 min incubation at room temperature, 600 μl of 70 % ethanol was added and the extraction was carried out according to the manufacturer’s purification protocol for total RNA. 600 μl of 70 % ethanol was added directly to Aptima sample aliquots without adding lysis solution. Total RNA was eluted in 50 μl of elution buffer and stored at −70 °C until reverse transcription was performed. From each FFPE sample four 20-μm sections were prepared and RNA was isolated using the RecoverAll total RNA isolation kit (Ambion, Austin, TX). Total RNA from cultured cells was isolated using the mirVana RNA isolation kit (Ambion). RNA concentrations were measured in NanoDrop instrument (Thermo Scientific, Wilmington, DE).

### MicroRNA real-time PCR assays

Array-format microRNA real-time PCR (rtPCR) miRNA assays were tailored according to the manufacturer’s design parameters (Exiqon) for HPV16-miR-H1, -H2, -H3, -H5 and -H6 (Table 1). Manufacturer-established assays were used for human endogenous RNU6B small nuclear RNA and spiked UniSp6 small RNA (Exiqon). The miRCURY LNA Universal RT microRNA PCR assays were run according to the instructions by the manufacturer (Exiqon). The reverse transcription (RT) reactions contained initially 10 ng of extracted RNA, 2 μl of 5× reaction buffer (including universal RT primer), 1 μl of enzyme mix, 0.5 μl of UniSp6 RNA spike-in template (75 amol) and water in a total volume of 10 μl. Later, in an attempt to increase the detection rate, a maximal volume (6.5 μl) of extracted RNA was used representing a range of 15–300 ng total RNA in a RT reaction. According to assay protocol, UniSp6 small RNA was added in the RT reaction, providing the samples with an internal control to confirm technical performance of the RT and rtPCR reactions. The RT reactions were incubated at 42 °C for 1 h followed by 95 °C for 5 min, and the resulting complementary DNAs (cDNAs) were stored at −70 °C unless rtPCR was performed the same day. For rtPCR reactions the cDNAs were diluted 1:100 in nuclease-free water. The diluted template was combined 1:1 with 2× ExiLENT SYBR Green master mix, and 10 μl of this reaction mix was added to each well. All rtPCR reactions were carried out in triplicates. All assays were run using the ABI 7500 real-time PCR instrument with 96-well format. In rtPCR, initial 10 min denaturation at 95 °C was followed by 40 amplification cycles (first seven Aptima samples) of 10 s at 95 °C and 1 min at 60 °C. For all other samples the number of cycles was raised to 60 in order to improve sensitivity. The assays were designed such that all miRNA assays from any individual sample were performed in one run in order to avoid repeated freezing and thawing. Non-template controls remained negative for all miRNA assays.

**Table 1:** HPV 16 encoded miRNA candidates [modified from Qian et al. (2013)]

miRNA name	Reference genome	Location	Annotation	Strand	Mature sequence
HPV16-miR-H1	NC_001526.2	2635–2716	E1	+	AGUGUAUGAGCUUAAUGAUAA
HPV16-miR-H2	FJ610147.1	56-1/7906–7851	LCR	−	AUGUGUAACCCAAAACGGUUUG
HPV16-miR-H3	NC_001526.2	518–642	LCR	+	CAACUGAUCUCUACUGUUA
HPV16-miR-H5	NC_001526.2	2471–2556	E1	+	GUAAAGCAUAGACCAUUG
HPV16-miR-H6	NC_001526.2	6684–6584	L1	–	AUCAACAACAGUAACAAA

### Target prediction for HPV16-miR-H6

HPV16-miR-H6 was a particularly interesting microRNA because it had by far the highest read counts in small RNA sequencing in our previous study (Qian et al. 2013). We therefore performed target prediction of H6 using the TargetScan Custom algorithm (Lewis et al. 2005) (Additional file 1: Table S1). The list of predicted target genes was screened by the DAVID 6.7 annotation tool (Dennis et al. 2003; da Huang et al. 2009) using default DAVID parameters for overrepresented biological themes (Additional file 2: Table S2).

## Results

We studied the expression of HPV encoded microRNA in a set of 52 cervical samples and four HPV containing cell lines. The clinical samples included 12 Aptima samples, four HC2 samples, 16 ThinPrep samples and 20 FFPE samples. Altogether 47 samples were hrHPV positive, three were hrHPV negative, and the HPV status of one sample could not be defined due to lack of sample material. One hrHPV positive Aptima sample was interpreted as invalid in the miRNA assays and was thus omitted from the analysis, resulting in 51 analysed clinical samples and four cell lines. All valid samples and their HPV status are described in Table 2. Histology, when available, is given for the actual sample (FFPE samples), or for a biopsy taken at the time of HPV sampling (Aptima, HC2, ThinPrep).

**Table 2:** Clinical samples and cell lines, HPV test results, histology at time of sampling, and microRNA assay results

N:o	Sample type	HC2	Aptima	LDR	Luminex	GeneXpert	Histology	H1	H2	H3	H5	H6	RNU6B	UniSp6
1^a^	Aptima	+	16				Normal	Neg	Neg	Neg	Neg	Neg	32	21
2^a^	Aptima	+	16				CIN3	Neg	Neg	Neg	Neg	Neg	33	21
3^a^	Aptima	+	16				CIN1	Neg	Neg	Neg	Neg	Neg	30	21
4^a^	Aptima	+	16				VAIN2	38 (1)	Neg	Neg	Neg	Neg	31	22
5^a^	Aptima	+	16				Normal	Neg	Neg	Neg	Neg	Neg	31	22
6^a^	Aptima	+	16				CIN1	Neg	Neg	Neg	Neg	Neg	34	22
7^a^	Aptima	+	16				Normal	Neg	Neg	Neg	Neg	Neg	32	21
8	Aptima	+	16				Carcinoma	Neg	Neg	Neg	Neg	Neg	32	22
9	Aptima	−	−				Normal	Neg	Neg	Neg	Neg	Neg	30	22
10	Aptima	+	16				CIN1	Neg	Neg	Neg	Neg	Neg	32	22
11	Aptima	−	−				Normal	Neg	Neg	Neg	Neg	Neg	28	21
12	HC2	+					N/A	Neg	Neg	Neg	Neg	51 (1)	24	21
13	HC2	+					Metaplasia	Neg	Neg	Neg	Neg	Neg	29	22
14	HC2	+					N/A	Neg	Neg	Neg	Neg	49 (1)	26	22
15	HC2	+					Normal	Neg	Neg	Neg	Neg	Neg	31	22
16	ThinPrep	+				hrHPV	CIN2	Neg	Neg	Neg	Neg	57 (2, var)	30	22
17	ThinPrep	+				16	CIN3	Neg	Neg	*44*	50 (2, var)	48 (2, var)	24	22
18	ThinPrep	+				18/45	Normal	Neg	Neg	Neg	52 (1)	Neg	28	22
19	ThinPrep	+				18/45	AIS	Neg	39 (1)	58 (1)	Neg	51 (1)	28	21
20	ThinPrep	+				hrHPV	CIN3	Neg	Neg	49 (2, var)	54 (1)	44 (2, var)	25	22
21	ThinPrep	+				hrHPV	Metaplasia	50 (1)	Neg	50 (3, var)	56 (1)	42 (3, var)	24	22
22	ThinPrep	+				16	CIN1	Neg	Neg	45 (1)	Neg	53 (1)	28	22
23	ThinPrep	+				16, hrHPV^b^	CIN1	Neg	Neg	58 (2, var)	Neg	52 (2, var)	27	22
24	ThinPrep	+				hrHPV	CIN2	Neg	39 (1)	Neg	Neg	Neg	33	23
25	ThinPrep	+				hrHPV	CIN1	Neg	Neg	50 (3, var)	Neg	48 (3, var)	27	22
26	ThinPrep	+				hrHPV	Condyloma	Neg	39 (1)	Neg	49 (1)	56 (1)	32	22
27	ThinPrep	+				16	CIN2	Neg	Neg	47 (3, var)	Neg	45 (3, var)	24	22
28	ThinPrep	+				18/45	Normal	Neg	Neg	Neg	Neg	Neg	31	23
29	ThinPrep	+				hrHPV	CIN1	Neg	37 (1)	55 (1)	Neg	57 (1)	29	23
30	ThinPrep	+				16	CIN3	50 (1)	39 (1)	45 (3, var)	48 (1)	45 (3, var)	25	22
31	ThinPrep	+				16	CIN2	Neg	Neg	50 (2, var)	Neg	47 (2, var)	27	22
32	FFPE	+		16, 18	16		SCC	Neg^c^	Neg	45 (2, var)	54 (1)	*48 (3, var)*	31	22
33	FFPE	−		6, 16			CIN3	52 (1)^c^	Neg^c^	47 (3, var)	48 (2, var)	*39 (3, var)*	33	22
34	FFPE	+		16, 18	16		AIS	42 (1)^c^	Neg^c^	46 (3, var)	51 (2, var)	*44 (3, var)*	33	21
35	FFPE	−		16, 18	16		CIN3	55 (1)^c^	57 (2, var)^c^	*45 (3, var)*	*42*	41 (3, var)	31	22
36	FFPE	+		16			Adenocarcinoma	Neg^c^	Neg^c^	*44 (3, var)*	47 (2, var)	42 (3, var)	32 (2)	21
37	FFPE	+		16, 18	16		SCC	57 (1)^c^	Neg^c^	46 (3, var)	47 (2, var)	*41*	33	21
38	FFPE	−		16	16, 31, 35		CIN3	56 (1)^c^	40 (1)	*47 (3, var)*	*47 (3, var)*	*40 (3, var)*	33	22
39	FFPE	N/A					Adenocarcinoma	Neg	Neg	*44 (3, var)*	45 (1)	46 (3, var)	33 (2)	22
40	FFPE	+		16, 18, 33			CIN3	Neg^c^	Neg	44 (3, var)	60 (1)	46 (3, var)	29	22
41	FFPE	−		16	45		AIS	48 (1)^c^	Neg	*41 (3, var)*	44 (2, var)	41 (3, var)	31	22
42	FFPE	+		16, 58	16, 33, 58		CIN1	Neg^c^	53 (1)	46 (3, var)	58 (1)	45 (3, var)	29	22
43	FFPE	−		16, 58	16, 18, 58		AIS	50 (1)^c^	Neg	*56 (3, var)*	Neg	58 (1)	34	23
44	FFPE	+		16, 18	33, 58		CIN1	Neg	44 (1)	45 (3, var)	52 (1)	*45 (3, var)*	33	22
45	FFPE	+		16, 18	16		AIS	Neg^c^	Neg^c^	56 (2, var)	Neg	53 (3, var)	38 (3, var)	24
46	FFPE	−		16	16		CIN2, p16+	55 (1)^c^	Neg	*57 (2)*	Neg	50 (3, var)	37 (2)	22
47	FFPE	+		16, 52	16, 35, 52, 56		CIN2, p16+	Neg^c^	Neg	45 (3, var)	Neg	47 (3, var)	32	22
48	FFPE	−		−	−		CIN2, p16+	52 (1)	Neg	59 (1)	Neg	50 (3, var)	37 (2)	25
49	FFPE	−		16, 18	16		Normal EE	Neg	Neg	Neg	Neg	Neg	37	22
50	FFPE	+		16	16		SCC	Neg^c^	Neg^c^	43 (1)	42 (1)	46 (3, var)	28	27
51b,c	FFPE	−		16	−		Normal SE	Neg	Neg	*44 (3, var)*	53 (2, var)	40 (3, var)	31	22
52	HPK IA						N/A	*39*	58 (1)	*38*	37	*34*	20	22
53	HPK II						N/A	*43 (3, var)*	Neg	*38 (3, var)*	39 (3, var)	*34*	26	21
54	CaSki						N/A	42 (3, var)	Neg	*40*	46 (3, var)	*34 (3, var)*	25	22
55	SiHa						N/A	49 (3, var)	Neg	*39 (3, var)*	48 (3, var)	*39 (3, var)*	28	21

*HC2* hybrid capture 2, *LDR* ligase detection reaction (LDR-PCR microarray), *H1*–*H6* HPV miRNAs, *CIN1*-*3* cervical intraepithelial neoplasia grade 1–3, *VAIN* vaginal intraepithelial neoplasia, *AIS* adenocarcinoma in situ, *SCC* squamous cell carcinoma, *EE* endocervical epithelium, *SE* squamous epithelium. *N/A* not applicable or not available. Numbers in parentheses tell the number of replicates giving signal; var, multiple signals were not obtained within one cycle. Italicised values are interpreted as true positive findings

^a^Amplification of 40 cycles was performed; for all other samples 60 cycles of amplification was used

^b^Positive both for HPV 16 and another high-risk HPV type(s)

^c^Expression was seen in some replicates in previous work

Human endogenous RNU6B small nuclear RNA and spiked UniSp6 were used as internal controls. RNU6B was amplified at cycles varying from Ct 20 to Ct 38. In five samples where RNU6B was amplified at high cycles, variation between the replicates was more than one cycle (Table 2). RNU6B was also amplified from all cell lines because they are of human origin. Of note, the input RNA amount was highly variable among the samples, which explains the variable Ct cycles of RNU6B amplification. UniSp6 spiked control was relatively uniformly amplified at cycles 21–27, mostly at Ct 21–22. Amplification of internal controls was unsuccessful from one hrHPV positive Aptima sample, which was thus interpreted as invalid and was omitted.

We have previously identified five putative HPV 16 encoded microRNAs (Qian et al. 2013), which are described in Table 1. HPV16-miR-H1 and HPV16-miR-H5 map to the positive strand within the E1 ORF, HPV16-miR-H2 and HPV16-miR-H3 to opposite strands of the LCR regulatory region, and HPV16-miR-H6 to the negative strand of the L1 ORF. In order to study the expression of putative HPV 16 encoded miRNAs, tailored LNA enhanced primer based assays were used because the assay design paradigms of looped-primer RT-PCR used in our previous study were not compatible with some candidate miRNA sequences. It is important to note that the assays were designed in the present work for the first time, and thus no comparison data from other studies are available. In the assay design we aimed at having similar parameters including Tm for all assays, and the assays were designed for mature miRNA sequences. In our material of clinical samples and cell lines, HPV 16 encoded miRNAs were amplified at high cycle counts (Table 2). Because low expression levels brought about large variation between replicates in some cases, the results were interpreted as follows: If three or two replicates gave signals within one amplification cycle, the sample was considered true positive for that particular miRNA (Table 2, italicised values). If signals from three or two replicates were obtained but they varied more than one cycle, or if a signal was obtained only from one replicate, the sample was not considered true positive, however the number of replicates giving a signal is given in Table 2.

HPV16-miR-H1 was detected in HPK IA and CaSki cells (3.6 % of all samples). None of the samples were interpreted true positive for HPV16-miR-H2, although positive signals were obtained from sporadic replicates. HPV16-miR-H3 was detected in one liquid cytology sample from a CIN3 patient, in eight paraffin samples (one normal squamous epithelium, one CIN2, two CIN3, two adenocarcinoma in situ, two adenocarcinoma samples), and in all four cell lines (23.6 %). HPV16-miR-H5 was detected in two CIN3 samples and in the HPK IA and HPK II cells (7.3 %). Expression of HPV16-miR-H6 was validated in one CIN1, two CIN3, two SCC and one AIS case, and in all four cell lines (18.2 %). All samples where miRNA expression was found were shown to be HPV 16 positive (Table 2), and the cell lines are known to contain HPV 16. HPV encoded miRNAs were not detected in any of the samples which remained negative in hrHPV detection and genotyping assays. Because of the rather small study size and the small number of samples per category, the expression levels were not normalized to RNU6B or UniSp6.

## Discussion

An important function of viral microRNAs is to modify viral gene expression, the cellular environment of virus replication, and the pathogenesis of viral diseases. Among human DNA tumor viruses at least the BK, JC and Merkel polyomaviruses encode miRNAs, which negatively regulate viral early gene expression with consequent escape from host immune attack and facilitation of viral replication (Seo et al. 2008, 2009; Lee et al. 2011). Papillomaviruses and polyomaviruses are both double-stranded DNA viruses causing persistent or latent infections and they share functional and molecular similarities, thus it is credible that papillomaviruses encode miRNAs of their own. Among the scarce scientific literature on the topic, detection of miRNAs encoded by human papillomaviruses has been unsuccessful by standard sequencing (Cai et al. 2006; Wang et al. 2014) or next generation sequencing (Lui et al. 2007). Computer predictions suggested, however, the existence of putative miRNA sequences encoded by mucosal and cutaneous HPV types (Gu et al. 2011). Our own studies have identified several putative HPV encoded microRNAs by SOLiD sequencing of small RNA libraries established from both human tissue samples and from HPV containing cell lines (Qian et al. 2013).

In the present work we report that expression of HPV encoded microRNAs in human cervical tissue or cell samples or in HPV containing cell lines is not infrequent, but amplification at high cycles suggests low expression levels of viral miRNAs. Viral miRNAs were found altogether in two normal/CIN1 cases and in eleven samples with severe histology (CIN2—carcinoma), suggesting that miRNA expression is found more often in high-grade lesions. Among human samples, the highest rate of miRNA signal was found in paraffin-embedded samples, confirming proper targeting of the HPV associated lesion by biopsy, and suggesting that the RNA extraction protocol used for these samples was successful. It also shows that microRNA can be successfully extracted and amplified from archival samples. Among other patient sample types, the only true HPV miRNA finding was HPV16-miR-H3 in a ThinPrep sample from a CIN3 patient. The amount of cells is much lower in cervical scrapings than in FFPE biopsy samples, which may explain poor detection of miRNAs, although RNU6B endogenous control was amplified at similar cycles from all sample types. Low detection rates may also be due to suboptimal extraction procedures for miRNA assay. Further, all HPV 16 harboring cell lines were found to express HPV microRNAs. Small RNAs used as controls were rigorously amplified at expected cycles, confirming the quality of the samples and the technical performance of the assay platform. Most of the FFPE samples were used in our previous validation study and were found positive for HPV16-miR-H1 or HPV16-miR-H2 by the looped-primer RT-PCR assay (Qian et al. 2013), but were not found positive using the LNA enhanced primer based assay in the present study. In the present work an LNA enhanced primer based assay was used because the design paradigms of the looped-primer RT-PCR assay were not compatible with some HPV miRNA sequences such as HPV16-miR-H6. Also, RNU6B was amplified at much higher cycles in the present study than in our previous work, suggesting differences in assay performance. According to the assay manufacturer, the LNA enhanced primer based assays used in the present work are more specific and, as suggested by our results, less sensitive than the looped-primer assays used in our previous work where identification of HPV microRNAs was presented (Qian et al. 2013). However, in our previous report, both TaqMan qPCR and in situ hybridization were successfully used to validate HPV16-miR-H1 and HPV16-miR-H2 expression. Different methods to study miRNA expression may typically produce different results, which are not always quantitatively comparable, and this complicates the evaluation of miRNA applicability for biomarker use. In our previous study, 100-fold differences in SOLiD small RNA sequencing data could not be reproduced in qPCR assays (Qian et al. 2013), suggesting that sequencing read counts cannot be used as a quantitative measure of miRNA expression. We further want to point out that the microRNA rtPCR assays designed for the present work have never been used before and therefore suboptimal assay design cannot be fully excluded.

Positive signals were most frequently obtained for HPV16-miR-H3, which maps to the positive strand within the LCR, and for HPV16-miR-H6 which maps to the negative strand of L1. In our previous miRNA sequencing study, the highest read counts by far were obtained for HPV16-miR-H6. Even low viral microRNA levels, however, may be sufficient to regulate viral replication and the expression of cellular target genes and thus have a role in the pathogenesis of viral diseases. Indeed, HPV16-miR-H2 has one predicted target sequence in the viral genome within the long control region upstream from replication origin in the HPV 16 genome (Qian et al. 2013).

Interestingly, all HPV 16 containing cell lines were found to express several HPV microRNAs, and they all expressed HPV16-miR-H3 and HPV16-miR-H6. The CaSki and SiHa cell lines have been established from cervical carcinoma tumors, whereas HPK IA and HPK II cell lines were originally established by transfecting HPV 16 DNA into foreskin primary keratinocytes (Dürst et al. 1987). The immortalized HPK cell lines are known to contain a number of chromosomal aberrations (Backsch et al. 2011). They represent clonal cell lines and thus every cell contains HPV 16, whereas in clinical samples HPV may be present in only a small proportion of cells. In our previous work, RNA sequencing produced 100-fold read counts for HPV16-miR-H6 as compared to other viral miRNAs. Target prediction for HPV16-miR-H6 yielded altogether 459 putative target genes (Additional file 1: Table S1), and functional annotation of the target genes revealed that most enriched biological process functions were cell adhesion and cell junction (Additional File 2: Table S2). Similar overrepresented functions were found among the predicted targets of human miRNAs which were differentially expressed due to HPV 16 E5 expression in our previous paper (Greco et al. 2011). Other potential functions among the targets were nuclear lumen, DNA binding, and calcium ion binding.

We conclude that the expression of human papillomavirus 16 encoded microRNAs was not infrequent but the expression levels were low in human cervical tissue and cell samples as well as in HPV 16 containing cell lines. Altogether microRNAs may have potential in the diagnosis, triage and follow-up of cervical disease, but cellular miRNAs regulated in HPV associated cervical disease hold more promise than viral miRNAs. However, frequent detection of viral miRNA, albeit at low levels, particularly in cell lines suggests that they may regulate viral replication and the expression of cellular target genes and thus have a role in the pathogenesis of viral diseases.

## Conclusions

Human papillomavirus 16 encoded microRNAs can be detected in cervical swabs, paraffin embedded tissue samples and in HPV containing cell lines. Because of low expression levels HPV encoded miRNAs are not well suited for biomarkers, but they may play a role in virus replication and in the pathogenesis of HPV associated diseases.

## Additional files

**Additional file 1: Table S1.** Prediction of HPV16-miR-H6 target genes using the TargetScan Custom algorithm (Lewis et al. 2005).

**Additional file 2: Table S2.** Annotation of predicted HPV16-miR-H6 target genes by DAVID annotation tool (Dennis et al. 2003; Huang et al. 2009).

## Abbreviations

AIS: adenocarcinoma in situ
cDNA: complementary DNA
CIN: cervical intraepithelial neoplasia
FFPE: formalin-fixed paraffin-embedded
HPV: human papillomavirus
hrHPV: high-risk human papillomavirus
miRNA: microRNA
qPCR: quantitative PCR
RT: reverse transcription
rtPCR: real-time PCR
SCC: squamous cell carcinoma
